# Effect of different modes of TPE on the prognosis of hypertriglyceridemic acute pancreatitis: a single-center retrospective study

**DOI:** 10.3389/fmed.2025.1712999

**Published:** 2026-01-05

**Authors:** Ruiting Li, Yaqi Ouyang, Yaohui Ming, Ruishan Yao, Huiling Guo, Yanli Wu, Jin Zhou, Yaowen Yuan, Haiyan Huang, Yin Yuan, You Shang

**Affiliations:** Department of Critical Care Medicine, Union Hospital, Tongji Medical College, Huazhong University of Science and Technology, Wuhan, Hubei, China

**Keywords:** centrifugal plasma exchange, hypertriglyceridemia, hypertriglyceridemic acute pancreatitis, membrane plasma exchange, therapeutic plasma exchange

## Abstract

**Background:**

The management and treatment of hypertriglyceridemia-induced acute pancreatitis (HTG-AP) are similar to those of other causes of AP and include fluid resuscitation, nutritional support, and pain control. Along with supportive care, treatments for HTG specifically include therapeutic plasma exchange (TPE) to reduce the level of serum triglyceride (TG) which are crucial for the treatment and prognosis of HTG-AP patients.

**Aim:**

The purpose of this study was to evaluate the effects of different TPE modes [centrifugal TPE (cTPE) and membrane TPE (mTPE)] on the prognosis and clinical benefit of HTG-AP.

**Methods:**

This observational cohort study was conducted on 49 patients with HTG-AP who presented between January 2021 and December 2024. The patients were divided into the cTPE group and mTPE group based on the mode they received for TPE. Data were collected from patients included clinical characteristics, biochemical indices, therapeutic outcomes, and adverse events.

**Results:**

The results showed that TPE significantly reduced the levels of serum TG and total cholesterol (TC). Between the two TPE groups, the reduction of TC level in the cTPE group was more prominent. After the first TPE, the TG level decreased by 66.16% in the cTPE group *vs* 65.36% in the mTPE group. Both modes of TPE resulted in a significant decrease in platelet levels (11.75% cTPE group, 23.89% mTPE group). However, both modes of TPE improved the coagulation dysfunction of HTG-AP patients, cTPE significantly reduced D-dimer levels and improved the PH of HTG-AP patients when compared to mTPE. Furthermore, there was a significant difference in the cost of a single TPE between the two groups, and the cost of cTPE was lower.

**Conclusion:**

This was the first study to compare the clinical benefits of cTPE and mTPE during the treatment of HTG-AP. cTPE showed a more significant advantage in improving coagulation function and pH, and the reduction of TC level in the cTPE group was more prominent. Additionally, the single cost of cTPE is lower. But there is insufficient evidence that cTPE is more beneficial for HTG-AP than mTPE, performing cTPE in combination with other therapies may be beneficial.

## 1 Background

The pathogenesis of acute pancreatitis (AP), which is characterized by severe abdominal pain, imaging changes in the pancreas, and high pancreatic enzymes, occurs mainly due to the inflammatory reaction caused by the digestion of pancreatic tissues after pancreatic enzymes are activated in the pancreas (1). This systemic inflammatory response can lead to multiple organ failure in severe cases and is called severe AP (1). Besides alcohol consumption and gallstones, hypertriglyceridemia (HTG) is the most common cause of AP.

In China, with changes in the diet structure and lifestyle of the population, the incidence of HTG-induced AP (HTG-AP) is increasing every year. The incidence of HTG-induced severe AP (HTG-SAP) is also increasing, resulting in high mortality (2). HTG has surpassed alcohol to become the second leading cause of AP, and the mortality of HTG-SAP is significantly greater than that of severe biliary pancreatitis (3). HTG-SAP generally occurs in patients with an underlying disorder of lipoprotein metabolism, obesity, pregnancy, intolerance, and other metabolic diseases (3).

The management and treatment of HTG-AP are similar to those of other causes of AP and include fluid resuscitation, nutritional support, and pain control. Along with supportive care, treatments for HTG specifically include therapeutic plasma exchange (TPE), hemofiltration, heparin, intravenous insulin, and anti-HTG medications (4). The level of HTG is an important risk factor for HTG-AP, and the risk of developing HTG-AP in moderate-to-severe HTG patients is significantly greater than that in mild HTG patients (5, 6). Additionally, the recurrence rate of AP caused by HTG is significantly greater than that caused by other etiologies, and the HTG-AP recurrence incidence is closely associated with high levels of serum triglyceride (TG) (7). HTG can also lead to pancreatic microvascular disease, impeding blood flow to the pancreas and leading to tissue damage. This may be responsible for more severe forms of AP and a greater risk of complications (8).

Therefore, detecting serum TG and taking active therapeutic measures to reduce the level of serum TG are crucial for the treatment and prognosis of HTG-AP patients. TPE is an effective way to rapidly reduce blood TG levels to below 11.3 mmol/L in patients. Some researchers have demonstrated the effectiveness of TPE in reducing TG levels in HTG-AP patients (9). However, some studies have shown that TPE does not have a greater clinical benefit than conventional treatment for AP (10). There is some debate about this phenomenon because the pathogenesis of HTG-AP is very complex, and further adjustment for confounders is needed. However, the ability of TPE to rapidly reduce TG and pancreatic enzymes has been recognized and is considered to be important in patients with severe AP (9, 11). Along with its effectiveness in the management of HTG-AP, TPE also has a favorable safety profile in patients with HTG-AP (11). In clinical practice, two modes of TPE are used, which include centrifugal TPE (cTPE) and membrane TPE (mTPE). The choice of TPE mode worldwide depends on local economics and preferences, but these two plasma exchange modes differ in terms of their efficiency of plasma exchange, choice of anticoagulation mode, and economic benefits (12, 13). No research group has studied the difference in HTG-AP prognosis between the two modes of TPE. This was the first study to compare the clinical benefits of cTPE and mTPE to provide evidence for clinicians to select the mode of TPE during the treatment of HTG-AP.

## 2 Methods

### 2.1 Study design and participants

This retrospective study was conducted at the Department of Critical Care Medicine (CCM) of Wuhan Union Hospital (Wuhan, China) from January 2021 to December 2024. All adult patients who were admitted to Department of CCM for HTG-AP and who required TPE during the study period were selected. The type of TPE was selected by their respective physicians at the time of treatment. The study was started after it was approved by the Ethics Committee of Union Hospital, Tongji Medical College, Huazhong University of Science [Clinical Trails: (2025)0324].

### 2.2 Criteria and definitions

The diagnostic criteria for AP followed the Revised Atlanta Definitions (14). The diagnostic criteria for HTG-AP were as follows: (1) met the diagnostic criteria for AP; (2) the serum TG level was greater than 11.3 mmol/L, or the TG value was between 5.65 and 11.3 mmol/L, but the venous blood was chylous blood, and AP caused by other causes was excluded (the normal range of serum TG level: <1.7 mmol/L).

According to the 2012 revision of the Atlanta Classification (14) of AP, patients were classified into mild acute pancreatitis (MAP) when they had no local or systemic complications or organ failure; moderately severe acute pancreatitis (MSAP) when they had transient (<48 h) organ failure or local or systemic complications; and SAP when they had organ failure lasting >48 h.

The inclusion criteria were as follows: (1) diagnosis of HTG-AP; (2) age: 18–85 years; (3) assessment of the first episode; and (4) serum TG detected within 24 h after admission at the Department of CCM.

The exclusion criteria were as follows: (1) AP due to other etiologies (cholelithiasis, alcohol, pregnancy, tumor disease, sphincter dysfunction, bacterial and viral infections, drugs, or toxins); (2) incomplete information; (3) allergies to plasma; (4) contraindications for TPE.

### 2.3 Grouping methods

Patients were divided into the cTPE group and mTPE group based on the mode of TPE they received. All patients with HTG-AP who met the inclusion criteria received basic support treatment after admission. The treatment included diet control, gastrointestinal decompression, enema, venous thromboembolism (VTE) prevention, inhibition of secretion of pancreatic enzymes, fluid resuscitation, proton pump inhibitors for gastric acid secretion, maintenance of electrolyte and acid–base balance, anti-infection, oxygen therapy and mechanical ventilation if necessary, nutritional support, fluid resuscitation, and other comprehensive treatments. We follow the American Society for Apheresis (ASFA) guidelines and recommend adjusting the PE treatment plan based on the patient’s specific condition. The plasma volume should be calculated according to 1–1.5 times the plasma volume per exchange, using the Kaplan formula: plasma volume = 0.065 * body weight (kg) * (1−hematocrit). However, owing to the shortage of blood resources at Wuhan Union Hospital, 1,500–2,500 mL of fresh frozen plasma was selected as the replacement fluid in both TPE modes; albumin and other colloidal fluids were not used as the replacement fluid. The first initiation of TPE was within 24–48 h after admission to the Department of CCM. The cTPE equipment used Blood Cell Separator (COM.TEC, COM.TEC advance, Fresenius Kabi AG); the mTPE equipment came from two different manufacturers. The anticoagulant regimen followed during plasma exchange was citrate anticoagulation *in vitro* or heparin systemic anticoagulation. Temporary central vein access (femoral vein or internal jugular vein) was established in all patients before TPE.

### 2.4 Data collection

All data were obtained from hospital electronic database records and patient medical records. During hospitalization, the following information was collected: age, sex, comorbidities, biochemical indices [total bilirubin (TBIL), alanine aminotransferase (ALT), aspartate transaminase (AST), serum creatinine (Scr), blood urea nitrogen (BUN), lipase (LPS), and amylase (AMY)], lipid indices [TG, total cholesterol (TC), and low-density lipoprotein (LDL)], infection indices [white blood cell (WBC) count, procalcitonin (PCT), C-reactive protein (CRP)], potential of hydrogen (PH), serum calcium, lactate (LAC), PaO_2_/FiO_2_ (P/F), red blood cell (RBC) count, platelet level, coagulation function [prothrombin time (PT), activated partial thromboplastin time (APTT), antithrombin III (AT III), fibrinogen, and D-dimer], PE-related data (times, amount of plasma replaced per TPE, and treatment time), PE adverse reactions (anemia, low blood pressure, platelet loss, electrolyte imbalance, and allergic reactions), Intensive Care Unit (ICU) length of hospital stay, total length of hospital stay, and hospital mortality.

### 2.5 Statistical analysis

The data were analyzed using GraphPad Prism 10 (GraphPad Software Inc., San Diego, CA, USA). Normally distributed data were expressed as the mean ± standard deviation (SD). The Chi-square test was conducted to determine differences between categorical variables. The Student’s *t*-test was conducted to determine differences between the groups when the data followed a normal distribution, and the Mann–Whitney *U*-test was conducted to determine differences between the groups when the data followed a nonnormal distribution. In this study, “*” denotes *p* < 0.05, which was considered statistically significant; “**” denotes *p* < 0.01; and “***” denotes *p* < 0.001.

## 3 Results

### 3.1 Characteristics of the included patients

**Figure 1:**
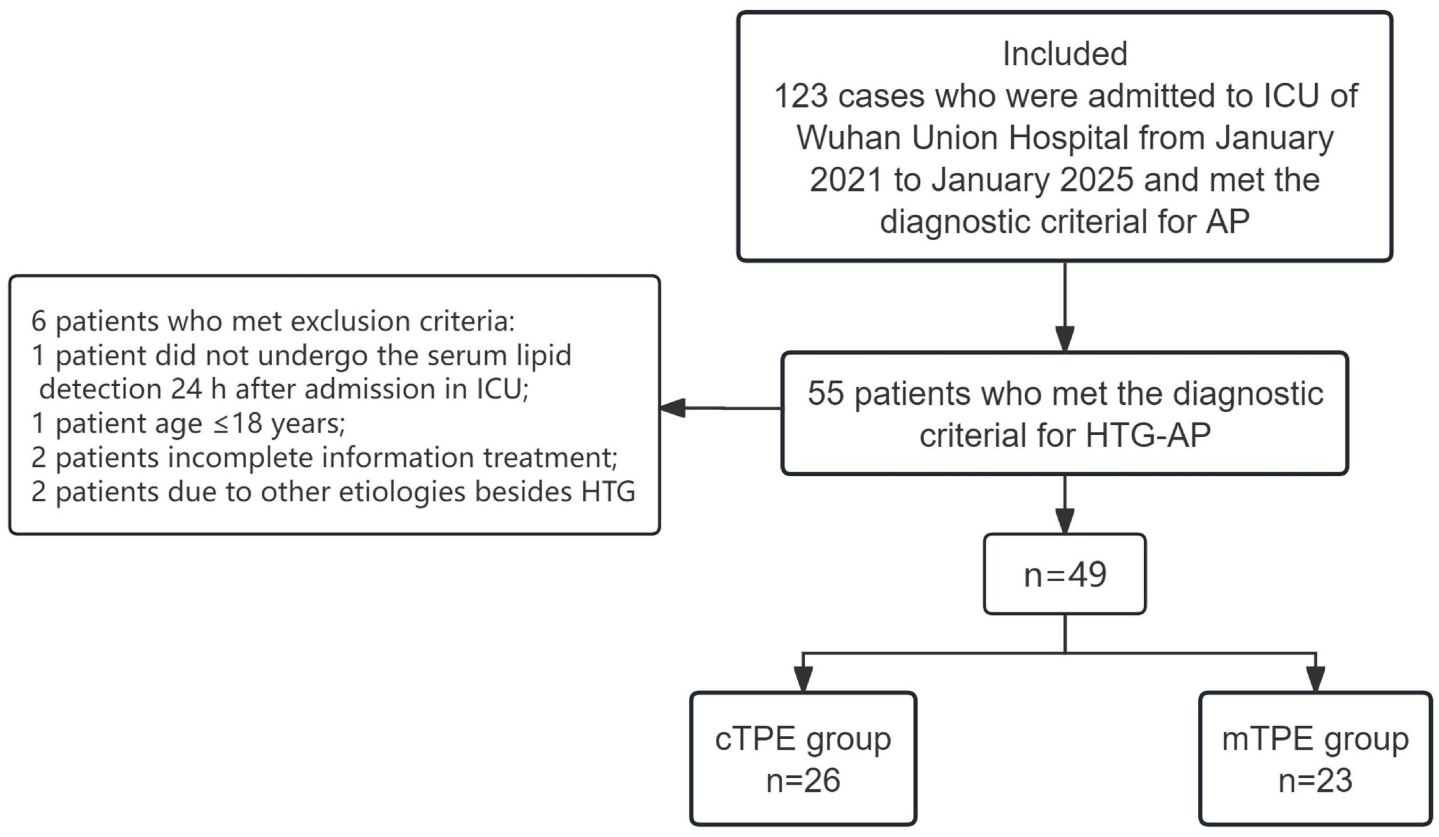
Flow chart of the patients included in the study. HTG, hypertriglyceridemia; cTPE, centrifugal TPE; mTPE, membrane TPE; AP, acute pancreatitis; HTG-AP, hypertriglyceridemia-induced AP; ICU, intensive care unit.

During the study period, 123 patients were diagnosed with AP at the Department of the CCM of Wuhan Union Hospital. Among these patients, 55 were diagnosed with HTG-AP. Six of these HTG-AP patients met the exclusion criteria: one patient did not undergo serum lipid detection 24 h after admission to the ICU, one patient was aged ≤18 years, two patients had incomplete treatment information, and two patients had etiologies other than HTG. In this study, 49 patients with HTG-AP were included. Moreover, cTPE was applied to 26 patients (cTPE group), and mTPE was applied to 23 patients (mTPE group) (Figure 1).

The mean age of the patients in the cTPE group was 39.9 ± 13.1 years, and the mean age of the patients who received mTPE was 38.5 ± 8.7 years (*p* = 0.676). Among the patients who underwent cTPE, 19.2% (*n* = 5) were female, and 80.8% (*n* = 21) were male. Among the patients who underwent mTPE, 21.7% (*n* = 5) were female, and 78.3% (*n* = 18) were male. No differences in age, sex, or comorbidities were found between the cTPE and mTPE groups (Table 1).

**Table 1:** Characteristics of the patients according to the therapeutic plasma exchange mode.

Variable	cTPE group (*n* = 26)	mTPE group (*n* = 23)	*p*
Age (mean ± SD)	39.9 ± 13.1	38.5 ± 8.7	0.676
Gender, *n* (%)			
Female	5 (19.2)	5 (21.7)	0.828
Male	21 (80.8)	18 (78.3)	
Comorbidity, *n* (%)			
Hypertension	4 (15.4)	3 (13.0)	0.815
Diabetes mellitus	5 (19.2)	3 (13.0)	0.559
Fatty liver	4 (15.4)	4 (17.4)	0.849
Gastrointestinal hemorrhage	1 (3.9)	0 (0)	0.342
Grades of severity, *n* (%)			
MAP	1 (3.9)	0 (0)	0.342
MSAP	2 (7.7)	2 (8.7)	0.898
SAP	23 (88.5)	21 (91.3)	0.743
Laboratory parameters (mean ± SD)			
LPS (U/L)	807.7 ± 643.1	696.0 ± 548.6	0.999
AMY (U/L)	510.5 ± 421.8	517.6 ± 424.2	0.899
PCT (ng/ml)	25.5 ± 43.7	34.7 ± 52.4	0.316
CRP (mg/L)	188.1 ± 117.0	258.3 ± 132.7	0.089
WBC (10^9^/L)	13.3 ± 7.9	12.3 ± 8.4	0.449
RBC (10^12^/L)	3.6 ± 0.9	2.9 ± 0.8	0.005^**^
Platelet (10^9^/L)	175.3 ± 103.2	130.6 ± 91.5	0.108
BUN (mmol/L)	13.0 ± 8.3	16.0 ± 9.9	0.235
Scr (μmol/L)	189.0 ± 139.3	244.5 ± 130.9	0.147
TBIL (μmol/L)	84.9 ± 97.1	104.6 ± 132.1	0.470
ALT (U/L)	79.8 ± 125.7	87.0 ± 149.0	0.889
AST (U/L)	285.4 ± 857.5	236.3 ± 383.9	0.988
Lactate (mmol/L)	2.9 ± 3.4	2.9 ± 2.9	0.623
P/F (mmHg)	186.5 ± 90.0	204.6 ± 86.7	0.518
CRRT/HP *n* (%)	4 (15.4)	3 (13.0)	0.815
Systematic complications, *n* (%)			
ARDS	22 (84.6)	20 (87.0)	0.815
AKI	18 (69.2)	15 (65.2)	0.765
AHI	15 (57.7)	12 (52.2)	0.698
Sepsis	23 (88.5)	21 (91.3)	0.743
Diabetic ketoacidosis	3 (11.5)	1 (4.4)	0.359

cTPE, centrifugal TPE; mTPE, membrane TPE, MAP: mild acute pancreatitis; MSAP, moderately severe acute pancreatitis; SAP, severe acute pancreatitis; CRRT: continuous renal replacement therapy; HP, hemoperfusion; ARDS, acute respiratory distress syndrome; AKI, acute kidney injury; AHI, acute hepatic injury; Scr, serum creatinine; BUN, blood urea nitrogen; LPS, Lipase; AMY, Amylase; WBC, white blood cell count; RBC, red blood cell count; PCT, procalcitonin; CRP, C-reaction protein; TBIL, total bilirubin; ALT, alanine aminotransferase; AST, aspartate transaminase; P/F, PaO_2_/FiO_2_; SD, standard deviation; **p < 0.01.

All patients were divided into the MAP group (*n* = 1), MSAP group (*n* = 4), and SAP group (*n* = 44). No significant difference was found in the number of patients with different severity levels of AP between the two groups (Table 1). Tables 1, 2 also shows that baseline LPS (*p* = 0.999), baseline AMY (*p* = 0.899), PCT (*p* = 0.316), CRP (*p* = 0.089), WBC (*p* = 0.449), platelet (*p* = 0.108), TBIL (*p* = 0.470), ALT (*p* = 0.889), AST (*p* = 0.988), BUN (*p* = 0.235), Scr (p = 0.147), PH (*p* = 0.322), serum calcium (*p* = 0.716), APTT (*p* = 0.246), PT (*p* = 0.109), fibrinogen (*p* = 0.624), D-dimer (*p* = 0.305), LAC (*p* = 0.623), and P/F (*p* = 0.518) at admission to the ICU were not significantly different between patients with different TPE modes. The HTG-AP patients treated with mTPE had significantly lower RBC counts than those in the group that received cTPE (*p* = 0.005). However, the overall difference in RBC levels between the two groups was not significant. No significant difference was found between the two groups in any of the systematic complications [acute respiratory distress syndrome (ARDS), acute kidney injury (AKI), acute hepatic injury (AHI), sepsis, and diabetic ketoacidosis].

**Table 2:** Comparisons of laboratory parameters with different therapeutic plasma exchange modes.

Variable	cTPE (*n* = 26)			mTPE (*n* = 23)			*p* (baseline value, cTPE vs. mTPE)
	Baseline	After 1st TPE	*p*	Baseline	After 1st TPE	*p*	
Laboratory parameters (mean ± SD)							
APTT (s)	43.9 ± 18.3	42.5 ± 18.2	0.243	52.4 ± 24.8	41.5 ± 11.9	0.014^*^	0.246
PT (s)	15.7 ± 2.3	15.1 ± 1.3	0.034^*^	18.7 ± 6.6	17.5 ± 5.9	0.002^**^	0.109
Fibrinogen (g/L)	6.6 ± 2.6	5.2 ± 1.6	0.001^**^	6.1 ± 2.7	4.0 ± 3.4	0.045^*^	0.624
D-dimer (mg/L)	7.3 ± 4.8	5.2 ± 2.9	0.002^**^	5.6 ± 4.0	6.7 ± 4.4	0.207	0.305
Platelet (10^9^/L)	175.3 ± 103.2	154.7 ± 84.6	0.007^**^	130.6 ± 91.5	99.4 ± 61.3	0.039^*^	0.108
TC (mmol/L)	11.0 ± 5.5	6.8 ± 5.5	0.0001^***^	10.2 ± 10.1	5.8 ± 3.1	0.004^**^	0.489
TG (mmol/L)	19.8 ± 14.7	6.7 ± 2.4	0.0001^***^	17.9 ± 10.1	6.2 ± 2.6	0.0001^***^	0.909
pH	7.33 ± 0.12	7.41 ± 0.06	0.0001^***^	7.34 ± 0.13	7.39 ± 0.07	0.099	0.322
Serum calcium (mmol/L)	2.0 ± 0.4	2.0 ± 0.3	0.543	1.9 ± 0.4	2.1 ± 0.2	0.124	0.716

cTPE, centrifugal TPE; mTPE, membrane TPE; TC, total cholesterol; TG, triglyceride; PT, prothrombin time; APTT, activated partial thromboplastin time; pH, potential of hydrogen; SD, standard deviation; *p < 0.05, **p < 0.01, ***p < 0.001.

### 3.2 Comparison of the effects of cTPE and mTPE on the decrease in serum lipid levels among HTG-AP patients

**Figure 2:**
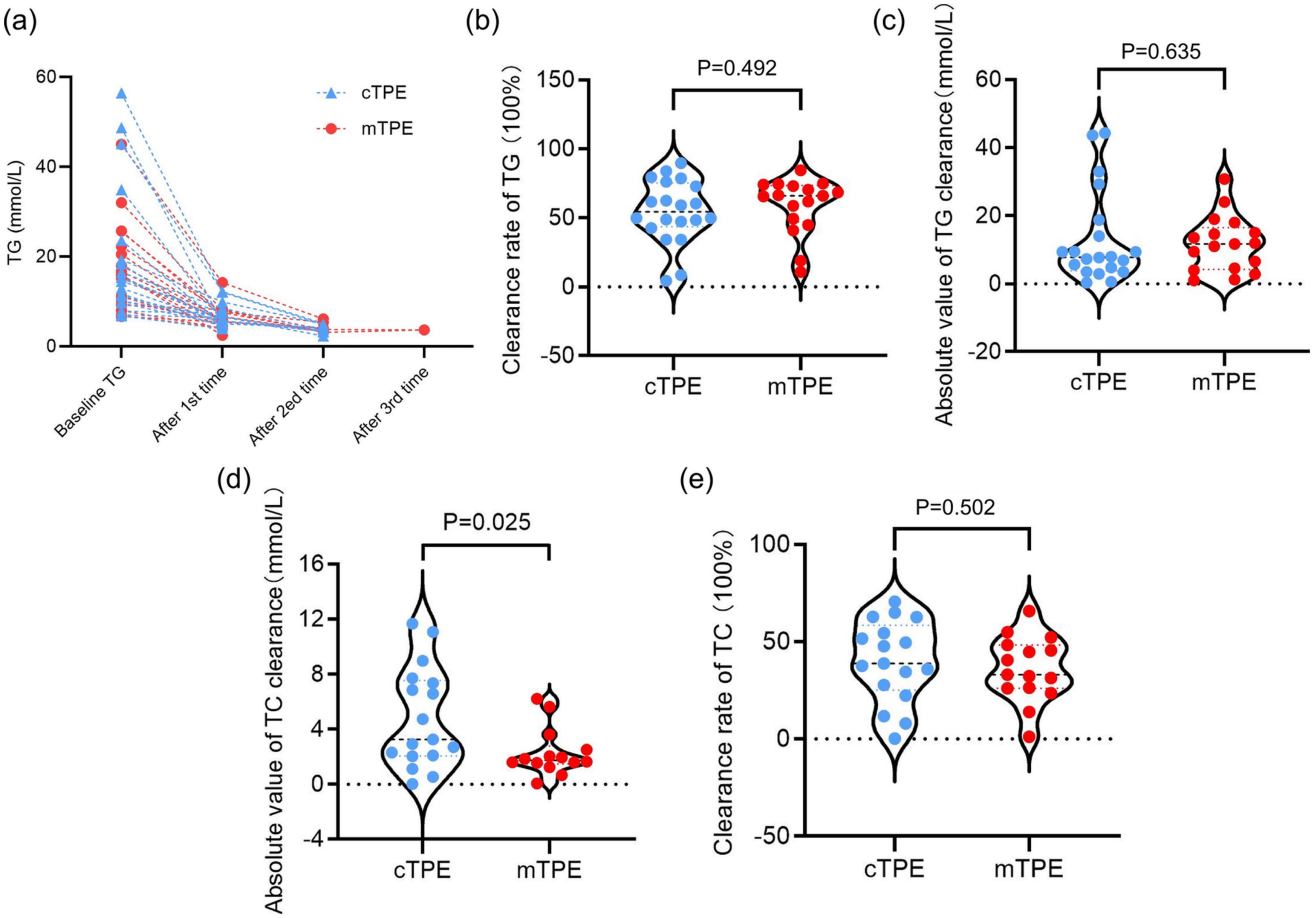
Effects of different TPE modes on serum TG and TC levels. **(a)** Baseline serum TG levels before TPE treatment and changes in TG levels after each TPE treatment in the two groups; **(b)** effects of two different modes of TPE on the clearance rate of TG; **(c)** effects of two different modes of TPE on the absolute value of TG clearance; **(d)** effects of two different modes of TPE on the clearance rate of TC; **(e)** effects of two different modes of TPE on the absolute value of TC clearance. ns, no significant difference.

A significant decrease in serum TG was recorded in patients treated with cTPE (1st *p* = 0.0001, 2nd *p* = 0.004) and mTPE (1st p = 0.0001, 2nd TPE *p* = 0.008) after the first and second TPE (Figure 2a, Table 2). After the first TPE, the TG level decreased by 66.16% in the cTPE group vs. 65.36% in the mTPE group. In the cTPE group, 45.0% of patients underwent a second plasmapheresis; in the mTPE group, 47.0% of patients underwent a second plasmapheresis, and 11.8% of patients underwent a third plasmapheresis due to a rebound in TG levels (Figure 2a). The clearance rates of TG were 54.6% ± 23.0% (cTPE) and 59.0% ± 20.2% (mTPE) (*p* = 0.492) in the first TPE session, but the difference between the two groups was not significant (Figure 2b). The absolute values of TG clearance in the cTPE group and mTPE group were 13.1 ± 13.6 mmol/L and 11.7 ± 8.2 mmol/L, respectively, in the first TPE session. However, no significant difference was found between the two groups (*p* = 0.635) (Figure 2c). A significant decrease in the serum TC level was recorded after the first occurrence of both cTPE and mTPE, with a significant difference in the cTPE group being more noticeable (*p* = 0.0001 vs. *p* = 0.004) (Table 2). By further comparing the absolute value of TC clearance, it is found that in cTPE group the decrease of TC level is more significant (4.8 ± 3.6 mmol/L vs. 2.3 ± 1.7 mmol/L, *p* = 0.025), but no significant difference was found between the two groups in the clearance rates of TC (*p* = 0.502) (Figures 2d,e).

### 3.3 Comparison of the effects of cTPE and mTPE on PH and serum calcium among HTG-AP patients

**Figure 3:**
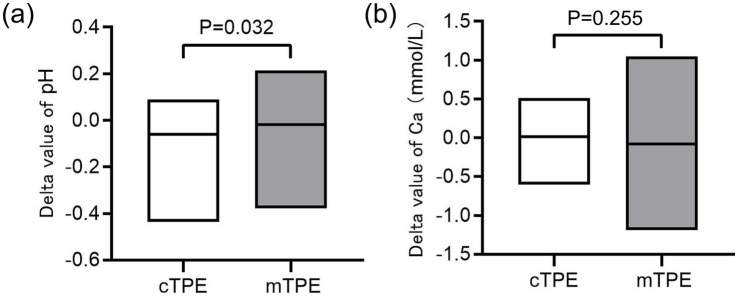
Comparing the effects of different TPE modes on pH and serum calcium. **(a)** Effects of two different modes of TPE on the delta value of pH; **(b)** Effects of two different modes of TPE on the delta value of serum calcium.

Due to the differences in the clearance efficiency of citrate and its metabolites between the two TPE modes, we analyzed the effects of different modes of TPE on citrate-related side effects: calcium ions and acid–base imbalance. As show in Figure 3, Supplementary Figure S1, after cTPE, the pH of patients improved significantly compared with the baseline value (*p* < 0.0001), but no significant difference was found after the mTPE (*p* = 0.322). When comparing the delta value of pH after the two TPE modes, there was a statistically significant difference (*p* = 0.032). However, when comparing the differences in serum calcium, Neither the comparison before and after different TPE modes (cTPE: *p* = 0.543; mTPE: *p* = 0.716) nor the comparison of delta value of serum calcium after the two TPE, there was no statistically significant difference (*p* = 0.255).

### 3.4 Comparison of the effects of cTPE and mTPE on coagulation function and platelet levels among HTG-AP patients

**Figure 4:**
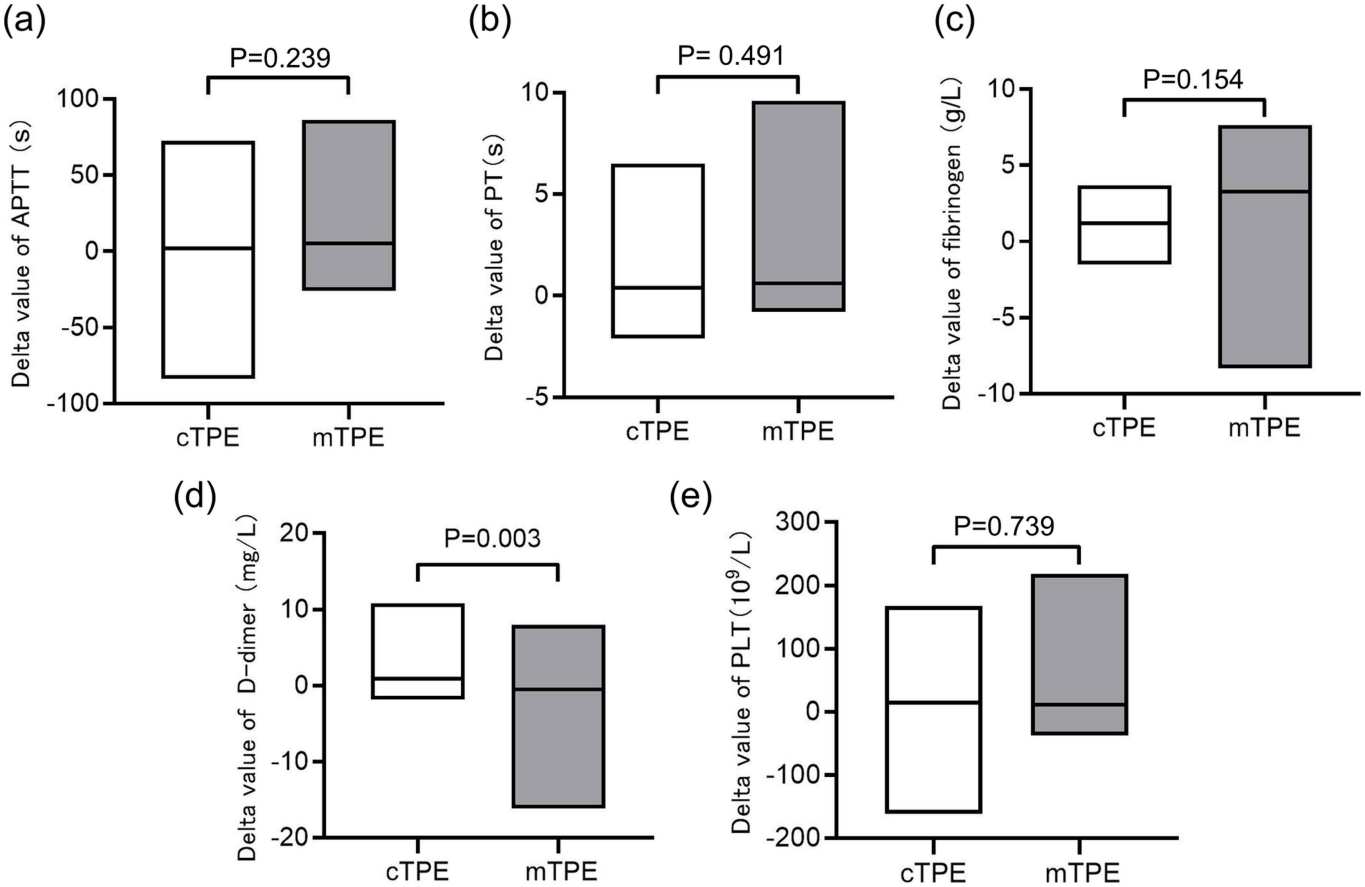
Comparing the effects of different TPE modes on platelet levels and coagulation function indicators. **(a)** Effects of two different modes of TPE on the delta value of APTT; **(b)** effects of two different modes of TPE on the delta value of PT; **(c)** effects of two different modes of TPE on the delta value of fibrinogen levels; **(d)** effects of two different modes of TPE on the delta value of D-dimer levels; **(e)** effects of two different modes of TPE on the delta value of platelet levels. ns, no significant difference.

We also recorded the effects of different TPE modes on coagulation function and platelet levels, which represent their safety and risk of bleeding complications. As shown in Table 2, Supplementary Figure S2, cTPE and mTPE significantly improved the PT of HTG-AP patients (*p* = 0.034, *p* = 0.002) and reduced the level of fibrinogen (*p* = 0.001, *p* = 0.045); the effect of cTPE on fibrinogen was more noticeable. Additionally, cTPE significantly reduced D-dimer levels (p = 0.002), no significant difference was found in the mTPE group (*p* = 0.207). Further comparison showed no statistical difference in the delta values of APTT, PT and fibrinogen changes between the two groups (*p* = 0.239, *p* = 0.491, *p* = 0.154), but there were statistical differences in the delta values of D-dimer changes (*p* = 0.003) (Figures 4a–d). Both modes of TPE resulted in a significant decrease in platelet levels (11.75% *p* = 0.007 cTPE group, 23.89% *p* = 0.039 mTPE group) (Supplementary Figure S3, Table 2). However, there was no statistical difference in the delta value of platelet level decrease between the two groups (*p* = 0.739) (Figure 4e).

### 3.5 Comparison of economic and clinical benefits of different TPE modes

The length of stay in the ICU was longer in the mTPE group than in the cTPE group (19.6 ± 19.2 days vs. 15.5 ± 17.5 days). However, no significant difference was found in the length of hospital stay or length of ICU stay (*p* = 0.785, *p* = 0.400). The mortality rate during hospitalization in patients who underwent mTPE was higher than that in patients who underwent cTPE (26.1% vs. 15.4%). However, no significant difference in mortality was found between the groups during hospitalization (*p* = 0.354) (Table 3). We also compared the differences in economic benefits between the two TPE modes. As shown in Table 3, Supplementary Figures S4, S5, a significant difference was also found in the cost of single TPE, the cost of cTPE is lower (6,763 ± 456.0 RMB vs. 7,408 ± 855.3 RMB, *p* = 0.0002). No significant difference was found between the two groups in total cost of TPE, hospitalization cost during ICU stay and total plasma volume (*p* = 0.571, *p* = 0.431, *p* = 0.999).

**Table 3:** Comparison of benefits of different therapeutic plasma exchange modes.

Variable	cTPE group (*n* = 26)	mTPE group (*n* = 23)	*p*
Length of hospital stay (days) (mean ± SD)	24.6 ± 16.6	24.7 ± 19.3	0.785
Length of ICU stay (days) (mean ± SD)	15.5 ± 17.5	19.6 ± 19.2	0.400
Total cost of TPE (RMB) (mean ± SD)	16,338 ± 13,233	14,331 ± 8,962	0.571
Cost per TPE (RMB) (mean ± SD)	6,763 ± 456.0	7,408 ± 855.3	0.0002^***^
Hospitalization costs during ICU stay (RMB 10,000) (mean ± SD)	35.9 ± 40.32	42.9 ± 37.8	0.431
Total plasma volume (mL) (mean ± SD)	4,052 ± 3,213	3,763 ± 2,545	0.999
Mortality, *n* (%)	4 (15.4)	6 (26.1)	0.354

PE, plasma exchange; cTPE, centrifugal TPE; mTPE, membrane TPE; ICU, intensive care unit; SD, standard deviation; RMB, Ren Min Bi; ***p < 0.001.

## 4 Discussion

In this study, the TG level was significantly lower in patients who underwent TPE, but no significant difference was found in either the TG clearance rate or the absolute value of TG clearance between the two TPE groups. A significant decrease was also found in serum TC level after the two modes of TPE were administered; among them, the significant difference in the cTPE group was more prominent. By further comparing the absolute value of TC clearance, it is found that in cTPE group the decrease of TC level is more significant. After cTPE, the pH of patients improved significantly compared with the baseline value, at the same time, there was a statistically significant difference on the delta value of pH between the two groups. Both modes of TPE resulted in a significant decrease in platelet levels. However, both modes of TPE improved the coagulation dysfunction of HTG-AP patients, including APTT, PT, fibrinogen, and D-dimer levels, to some extent. Some differences were found between the two groups, and mTPE did not significantly reduce D-dimer levels in HTG-AP patients. Further comparison showed there were statistical differences in the delta values of D-dimer changes between the two groups. Furthermore, there was a significant difference in the cost of a single TPE between the two groups, and the cost of cTPE was lower.

The pathogenesis of HTG-AP is complex, the incidence of complications is high, severe cases are often complicated with multiple organ failure, and the prognosis is poor. The most accepted mechanism of HTG-AP is the hydrolysis of TG by pancreatic enzymes into free fatty acids, which in turn triggers an inflammatory cascade, resulting in damage to acinar cells and tissue capillaries. High levels of TG can cause acute fat embolism in pancreatic arterioles and microcirculation and aggravate ischemic necrosis of the pancreas (10). Besides administering standard therapy for pancreatitis, reducing serum TG levels quickly is important for the early treatment of HTG-AP (15). Specific treatments, including heparin and insulin, as well as TPE, are administered to reduce TG levels (16). Compared with conventional treatment, TPE did not improve the complications, organ support treatment, surgical rate of HT-GAP, nor reduced the hospital stay and mortality (10), but TPE still is a method that rapidly reduces plasma TG levels. Although there are several observational and retrospective studies on PE in the treatment of HTG-AP, the conclusions are still controversial. The treatment of AP is a very complex process that may involve supportive treatment of multiple organs, and it is not an independent factor of TPE alone that can improve the prognosis of HTG-AP patients. However, researchers have determined the role of TPE in rapidly reducing TG and pancreatic enzymes (17). PE not only quickly and effectively removes excess TG, pancreatic enzymes, phospholipase A2, and proinflammatory cytokines in HTG-AP patients but also supplements the lack of coagulation factors, thus stabilizing the internal environment of the patients and correcting the excessive release of proinflammatory cytokines and the imbalance between proinflammatory and anti-inflammatory cytokines. The inflammatory cascade is blocked, and the development of pancreatitis is controlled (18).

There are two modes of TPE, including mTPE and cTPE, which are both well-established techniques (19). In TPE using the centrifugal technique, the plasma separated out is discarded, and RBCs and fresh healthy plasma are mixed or returned to the patient. The membrane plasma separation technique allows the selective removal of undesired proteins or macromolecules (20). The TPE efficiency and plasma extraction rate of the centrifugal technique are greater than those of the membrane separation technique; the cTPE technique requires less time than the mTPE method (12, 21). Additionally, the main difference between mTPE and cTPE is the anticoagulation method, ease of use, and complications (21). The choice of TPE mode worldwide depends on local economics and preferences. No prospective randomized controlled trials (RCTs) or retrospective studies comparing the therapeutic effects of mTPE and cTPE on HTG-AP are available.

We compared the effects of two TPE modes in treating patients with HTG-AP. A significant decrease in serum TG levels was found in patients after one session of mTPE or cTPE. However, no significant difference in the clearance rates of TG or the absolute value of TG clearance was found between the cTPE and mTPE groups. Along with the serum TG level, the serum TC level is related to the pathogenesis of HTG-AP (22, 23). Hong et al. (22) reported that the TC level within 24 h of admission had a U-shaped relationship with the severity of AP. High TC levels were associated with a significantly greater risk of severe AP and a longer hospital stay than moderate TC levels. Sun et al. (23) reported that HTG, hypercholesterolemia, and obesity serve as risk factors for AP, particularly among young and middle-aged men. TG and TC act as mediating factors between body mass index and the risk of developing AP. Our results revealed that both TPE modes reduced serum TG and TC faster and more effectively, and the cTPE mode had a greater advantage in reducing the level of serum TC in HTG-AP patients. These results revealed the advantages of cTPE in reducing TC and TG levels and controlling the progression of HTG-AP.

We also compared the effects of the two TPE modes on platelet and coagulation function, which indicates their safety. Some studies have reported that TPE increases platelet count and improves coagulation function in sepsis (24), while TPE was found to decrease platelet count in patients with liver failure (25). Hafer et al. (26) designed a randomized, prospective, paired crossover trial to compare mTPE and cTPE and reported that platelet loss caused by cTPE was only about half of that caused by mTPE. This study highlighted the advantage of the effect of cTPE on platelets. However, in our study, neither TPE mode showed an advantage in protecting platelets, and the level of platelets in blood decreased significantly after applying both plasmapheresis modes. The difference was more significant in the cTPE group, and the decrease in the platelet count was more prominent. This might be related to the disease status of the patients; SAP is easily associated with sepsis. Sepsis and excessive inflammation often affect platelet levels, and thrombocytopenia is very common in sepsis, with an incidence of > 55% in patients with sepsis (27). Regarding the effect on coagulation function, previous studies focused on the single factor of plasmapheresis (24). Plasma exchange may improve diffuse intravascular coagulation in patients by improving endothelial function (24). A prospective observational study revealed that in patients receiving warfarin treatment and undergoing TPE simultaneously, after a single TPE, the international normalized ratio (INR) was about twice that before the exchange, and the plasma levels of fibrinogen and coagulation factor II decreased by about 65 and 60%, respectively (28). Our study revealed that both cTPE and mTPE significantly improved the PT of HTG-AP patients, reduced the levels of fibrinogen, and that the effect of cTPE on fibrinogen was more noticeable. Additionally, cTPE can significantly reduce D-dimer levels. We speculate that this might be related to the easier formation of microthrombi in the membrane plasma separator. This was the first clinical study to compare the effects of mTPE and cTPE on the coagulation function of HTG-AP patients. This finding also revealed the advantages of cTPE in improving the coagulation function in HTG-AP patients.

We analyzed the effects of different modes of TPE on calcium ions and pH. This represents the influence of different TPE mode on citrate-related side effects. After cTPE, the pH of patients improved significantly compared with the baseline value. When further comparing the delta value of pH of cTPE with that of mTPE mode, the change of pH after cTPE is obvious. This is related to the high clearance rate of citrate and its metabolites in cTPE. The clearance rate of citrate in cTPE is up to 80%. Hence, the incidence of citrate-induced toxic reactions and metabolic alkalosis is markedly mitigated (29). Nevertheless, current clinical studies have reported that the routine use of low-dose RCA during mTPE is safe and effective and can be widely used in clinics (30). Due to the selection of citrate local anticoagulation and systemic anticoagulation regimens, calcium ion abnormalities may also occur during or after TPE. We found there are no significant difference of serum calcium after the two TPE.

We also found that the length of stay in the ICU, length of hospital stay, total cost of TPE, hospitalization costs during ICU stay, total plasma volume and mortality during hospitalization did not differ significantly between the two TPE groups. This occurred mainly because the pathogenesis of AP, especially SAP, is extremely complex and often involves multiple organ function damage and the possibility of septic shock; thus, it is not a single independent factor that can improve the prognosis of patients. This requires comprehensive treatment, even organ replacement therapy such as continuous renal replacement therapy (CRRT), extracorporeal membrane oxygenation, etc. (4). Nevertheless, we still found that a significant difference in the cost of single TPE, the cost of cTPE is lower. This is mainly related to the low cost of consumables required by centrifugal plasma separation equipment, cTPE eliminates the need for an expensive plasma separator. This reflected the difference in economic benefits between the membrane and the centrifugal method in treating HTG-AP, cTPE has more advantages in economic benefits.

This study had several limitations. This was a single-center retrospective study, and the number of patients in each group was small. Not all patients underwent all biochemical tests before and after plasmapheresis, and some data were missing for some patients. Moreover, owing to the shortage of blood resources in our hospital, the volume of plasma exchanged each time was relatively small, and it was not conducted according to 1–1.5 times the plasma volume per exchange, which probably led to deviations in the results. One more point that needs attention is that there are differences in the baseline levels of RBC counts between the two groups. The observed differences in outcomes could be influenced by this baseline discrepancy and that future prospective studies will employ statistical techniques to adjust for such confounders. Prospective, multicenter studies with large patient populations are needed to validate the differences between the two modes of TPE. HTG-AP is a very complex disease, and more confounding factors need to be considered to eliminate research bias.

## 5 Conclusion

There is insufficient evidence that cTPE is more beneficial for HTG-AP than mTPE, performing TPE along with combination therapy may be beneficial, as an early and rapid decrease in plasma TC and TG levels may affect clinical outcomes and the emergence of related complications. In this retrospective study, cTPE showed a more significant advantage in improving coagulation function and PH, which was associated with an improved prognosis in patients with acid–base imbalance, bleeding and thrombosis-related complications. However, we still need further prospective studies with large sample sizes to verify this conclusion and the differences between the two models. Additionally, the single cost of cTPE is lower, which highlights the economic benefit advantage of cTPE.

## Data Availability

The data analyzed in this study is subject to the following licenses/restrictions: It can be provided when requesting to the corresponding author. Requests to access these datasets should be directed to Prof. Ruiting Li 498676772@qq.com.

## Supplementary material

SUPPLEMENTARY FIGURE S1 Effects of different TPE modes on PH and serum calcium. **(a** and **c)** Change in PH after two different modes of TPE; **(b** and **d)** Change in serum calcium after two different modes of TPE. ns: no significant difference.

SUPPLEMENTARY FIGURE S2 Effects of different TPE modes on coagulation function indicators. **(a,b)** Change in APTT after two different modes of TPE; **(c,d)** change in PT after two different modes of TPE; **(e,f)** Changes in fibrinogen levels after two different modes of TPE; **(g,h)** changes in D-dimer levels after two different modes of TPE. ns, no significant difference.

SUPPLEMENTARY FIGURE S3 Effects of different TPE modes on platelet levels. **(a)** Changes in platelet levels after cTPE treatment; **(b)** changes in platelet counts after mTPE treatment.

SUPPLEMENTARY FIGURE S4 The benefit comparison of different TPE modes. **(a)** Total cost comparison of different TPE modes; **(b)** single cost comparison of different TPE modes; **(c)** hospitalization cost comparison during ICU stay of different TPE modes. ns, no significant difference.

SUPPLEMENTARY FIGURE S5 Comparison of total plasma volume and single plasma volume in different TPE modes.

## Glossary

AP: acute pancreatitis
HTG: hypertriglyceridemia
HTG-AP: HTG-induced AP
HTG-SAP: HTG-induced severe AP
TPE: therapeutic plasma exchange
TG: triglyceride
cTPE: centrifugal TPE
mTPE: membrane TPE
CCM: Critical Care Medicine
MAP: mild acute pancreatitis
MSAP: moderately severe acute pancreatitis
VTE: venous thromboembolism
TBIL: total bilirubin
ALT: alanine aminotransferase
AST: aspartate transaminase
Scr: serum creatinine
BUN: blood urea nitrogen
LPS: lipase
AMY: and amylase
TC: total cholesterol
LDL: low-density lipoprotein
WBC: white blood cell
PCT: procalcitonin
CRP: C-reactive protein
LAC: lactate
P/F: PaO_2_/FiO_2_
RBC: red blood cell
PT: prothrombin time
APTT: activated partial thromboplastin time
AT III: antithrombin III
ICU: Intensive Care Unit
SD: standard deviation
ARDS: acute respiratory distress syndrome
AKI: acute kidney injury
AHI: acute hepatic injury
RCTs: randomized controlled trials
INR: international normalized ratio
CRRT: continuous renal replacement therapy
pH: potential of hydrogen
ASFA: American Society for Apheresis

## References

[1] Crockett SD Wani S Gardner TB Falck-Ytter Y Barkun AN American Gastroenterological Association Institute Clinical Guidelines Committee. American Gastroenterological Association Institute guideline on initial management of acute pancreatitis. Gastroenterology. (2018) 154:1096–101. doi: 10.1053/j.gastro.2018.01.032, 29409760

[2] Yang AL McNabb-Baltar J. Hypertriglyceridemia and acute pancreatitis. Pancreatology. (2020) 20:795–800. doi: 10.1016/j.pan.2020.06.005, 32571534

[3] Carr RA Rejowski BJ Cote GA Pitt HA Zyromski NJ. Systematic review of hypertriglyceridemia-induced acute pancreatitis: a more virulent etiology? Pancreatology. (2016) 16:469–76. doi: 10.1016/j.pan.2016.02.011 27012480

[4] Gligorijevic N Stefanovic-Racic M Kershaw EE. Medical management of hypertriglyceridemia in pancreatitis. Curr Opin Gastroenterol. (2023) 39:421–7. doi: 10.1097/MOG.0000000000000956, 37421386

[5] Qiu L Sun RQ Jia RR Ma XY Cheng L Tang MC . Comparison of existing clinical scoring systems in predicting severity and prognoses of hyperlipidemic acute pancreatitis in Chinese patients: a retrospective study. Medicine (Baltimore). (2015) 94:e957. doi: 10.1097/MD.0000000000000957, 26061329 PMC4616466

[6] Xenoulis PG Cammarata PJ Walzem RL Suchodolski JS Steiner JM. Serum triglyceride and cholesterol concentra-tions and lipoprotein profiles in dogs with naturally occurring pancreatitis and healthy control dogs. J Vet Intern Med. (2020) 34:644–52. doi: 10.1111/jvim.15715, 32012351 PMC7097643

[7] Ding L Guan L Li X Xu X Zou Y He C . Recurrence for patients with first episode of hypertriglyceridemia-induced acute pancreatitis: a prospective cohort study. J Clin Lipidol. (2023) 17:94–102. doi: 10.1016/j.jacl.2022.11.006 36697323

[8] Mosztbacher D Hanák L Farkas N Szentesi A Mikó A Bajor J . Hypertriglyceridemia-induced acute pancreatitis: a prospective, multicenter, international cohort analysis of 716 acute pancreatitis cases. Pancreatology. (2020) 20:608–16. doi: 10.1016/j.pan.2020.03.018, 32402696

[9] Cao L Chen Y Liu S Huang W Wu D Hong D . Early plasmapheresis among patients with hypertriglyceridemia-associated acute pancreatitis. JAMA Netw Open. (2023) 6:e2320802. doi: 10.1001/jamanetworkopen.2023.20802, 37378979 PMC10308255

[10] Lin Y Yu S Wu X Huang L Huang S Huang Y . Clinical analysis of the therapeutic effect of plasma exchange on hypertriglyceridemic acute pancreatitis: a retrospective study. Transfusion. (2022) 62:72–81. doi: 10.1111/trf.16724, 34735720

[11] Kandemir A Co¸skun A Yava so˘glu I Bolaman Z Ünübol M Ya sa MH . Therapeutic plasma exchange for hypertriglyceridemia induced acut pancreatitis: the 33 cases experience from a tertiary reference center in Turkey. Turk J Gastroenterol. (2018) 29:676–83. doi: 10.5152/tjg.2018.17627 30289402 PMC6284677

[12] Keklik M Çelik S Yıldızhan E. Comparison of centrifugal and membrane filtration modalities on therapeutic plasma exchange. J Clin Apher. (2022) 37:217–22. doi: 10.1002/jca.21961, 34978347

[13] Kes P Janssens ME Bašić-Jukić N Kljak M. A randomized crossover study comparing membrane and centrifugal therapeutic plasma exchange procedures. Transfusion. (2016) 56:3065–72. doi: 10.1111/trf.13850, 27704559

[14] Banks PA Bollen TL Dervenis C Gooszen HG Johnson CD Sarr MG . Classification of acute pancreatitis—2012: revision of the Atlanta classification and definitions by international consensus. Gut. (2013) 62:102–11. doi: 10.1136/gutjnl-2012-302779, 23100216

[15] Lin XY Zeng Y Zhang ZC Lin ZH Chen LC Ye ZS. Incidence and clinical characteristics of hypertriglyceridemic acute pancreatitis: a retrospective single-center study. World J Gastroenterol. (2022) 28:3946–59. doi: 10.3748/wjg.v28.i29.3946, 36157550 PMC9367230

[16] Guo YY Li HX Zhang Y He WH. Hypertriglyceridemia-induced acute pancreatitis: progress on disease mechanisms and treatment modalities. Discov Med. (2019) 27:101–9. 30939294

[17] Padmanabhan A Connelly-Smith L Aqui N Balogun RA Klingel R Meyer E . Guidelines on the use of therapeutic apheresis in clinical practice-evidence-based approach from the writing Committee of the American Society for apheresis: the eighth special issue. J Clin Apher. (2019) 34:171–354. doi: 10.1002/jca.21705, 31180581

[18] Fonseca-González G Alamilla-Sánchez M García-Macas V Herrera-Acevedo J Villalobos-Brito M Tapia-Rangel E . Impact of plasmapheresis on severe COVID-19. Sci Rep. (2023) 13:163. doi: 10.1038/s41598-022-25930-8, 36599875 PMC9812351

[19] Puppe B Kingdon EJ. Membrane and centrifugal therapeutic plasma exchange: practical difficulties in anticoagulating the extracorporeal circuit. Clin Kidney J. (2014) 7:201–5. doi: 10.1093/ckj/sft163, 25852872 PMC4377769

[20] Sergent SR Ashurst JV. Plasmapheresis In: StatPearls. Treasure Island (FL): StatPearls Publishing (2025). 32809401

[21] Ahmed S Kaplan A. Therapeutic plasma exchange using membrane plasma separation. Clin J Am Soc Nephrol. (2020) 15:1364–70. doi: 10.2215/CJN.12501019, 32312791 PMC7480555

[22] Hong W Zimmer V Basharat Z Zippi M Stock S Geng W . Association of total cholesterol with severe acute pancreatitis: a U-shaped relationship. Clin Nutr. (2020) 39:250–7. doi: 10.1016/j.clnu.2019.01.022, 30772093

[23] Sun Q Du L Ren Q Zhu G Zhang B Su A . Hypertriglyceridemia, hypercholesterolemia, body mass index, and the risk of acute pancreatitis. Dig Dis Sci. (2024) 69:3413–25. doi: 10.1007/s10620-024-08493-8, 38987445

[24] Weng J Chen M Fang D Liu D Guo R Yang S. Therapeutic plasma exchange protects patients with Sepsis-associated disseminated intravascular coagulation by improving endothelial function. Clin Appl Thromb Hemost. (2021) 27:10760296211053313. doi: 10.1177/10760296211053313, 34775801 PMC8597066

[25] Maheshwari A Bajpai M Patidar GK. Effects of therapeutic plasma exchange on liver function test and coagulation parameters in acute liver failure patients. Hematol Transfus Cell Ther. (2020) 42:125–8. doi: 10.1016/j.htct.2019.05.003, 31387798 PMC7248502

[26] Hafer C Golla P Gericke M Eden G Beutel G Schmidt JJ . Membrane versus centrifuge-based therapeutic plasma exchange: a randomized prospective crossover study. Int Urol Nephrol. (2016) 48:133–8. doi: 10.1007/s11255-015-1137-3, 26531062 PMC5360823

[27] Leone M Nielsen ND Russell L. Ten tips on sepsis-induced thrombocytopenia. Intensive Care Med. (2024) 50:1157–60. doi: 10.1007/s00134-024-07478-5, 38739278

[28] Zantek ND Morgan S Zantek PF Mair DC Bowman RJ Aysola A. Effect of therapeutic plasma exchange on coagulation parameters in patients on warfarin. J Clin Apher. (2014) 29:75–82. doi: 10.1002/jca.21294, 24000079

[29] Coirier V Lesouhaitier M Reizine F Painvin B Quelven Q Maamar A . Tolerance and complications of therapeutic plasma exchange by centrifugation: a single center experience. J Clin Apher. (2022) 37:54–64. doi: 10.1002/jca.21950, 34786746

[30] Yuan F Li Z Li X Liu H. Application of regional citrate anticoagulation in membrane therapeutic plasma exchange. Int Urol Nephrol. (2020) 52:2379–84. doi: 10.1007/s11255-020-02581-0, 32740788

