# Supplementary figures and images for: Effect of different modes of TPE on the prognosis of hypertriglyceridemic acute pancreatitis: a single-center retrospective study
Source: Front Med (Lausanne). 2026 Jan 5;12:1712999. doi: 10.3389/fmed.2025.1712999 (PMC12813003; doi:10.3389/fmed.2025.1712999)

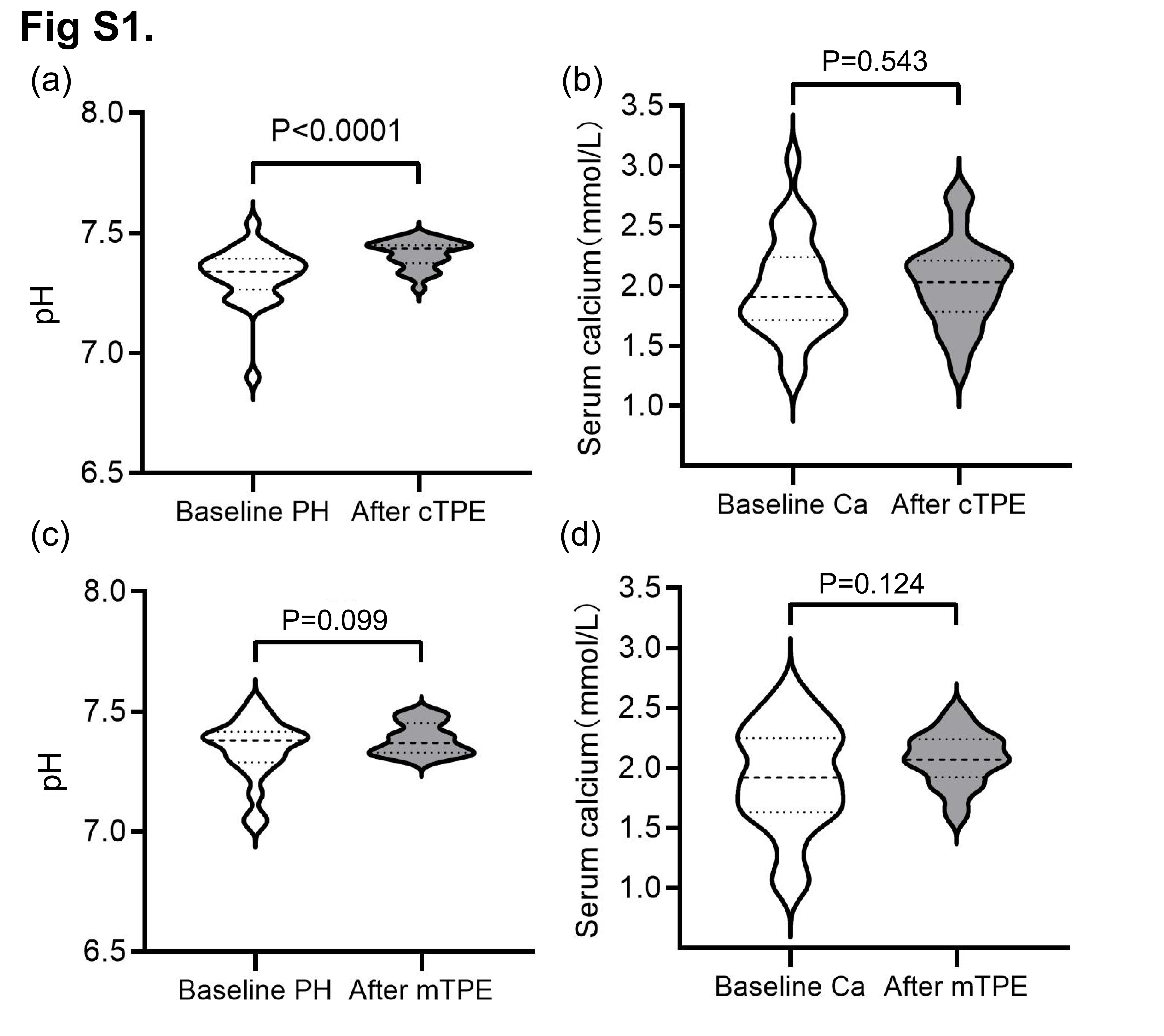

Supplement: SUPPLEMENTARY FIGURE S1 — Effects of different TPE modes on PH and serum calcium. (a and c) Change in PH after two different modes of TPE; (b and d) Change in serum calcium after two different modes of TPE. ns: no significant difference. [file Image_1.jpeg]

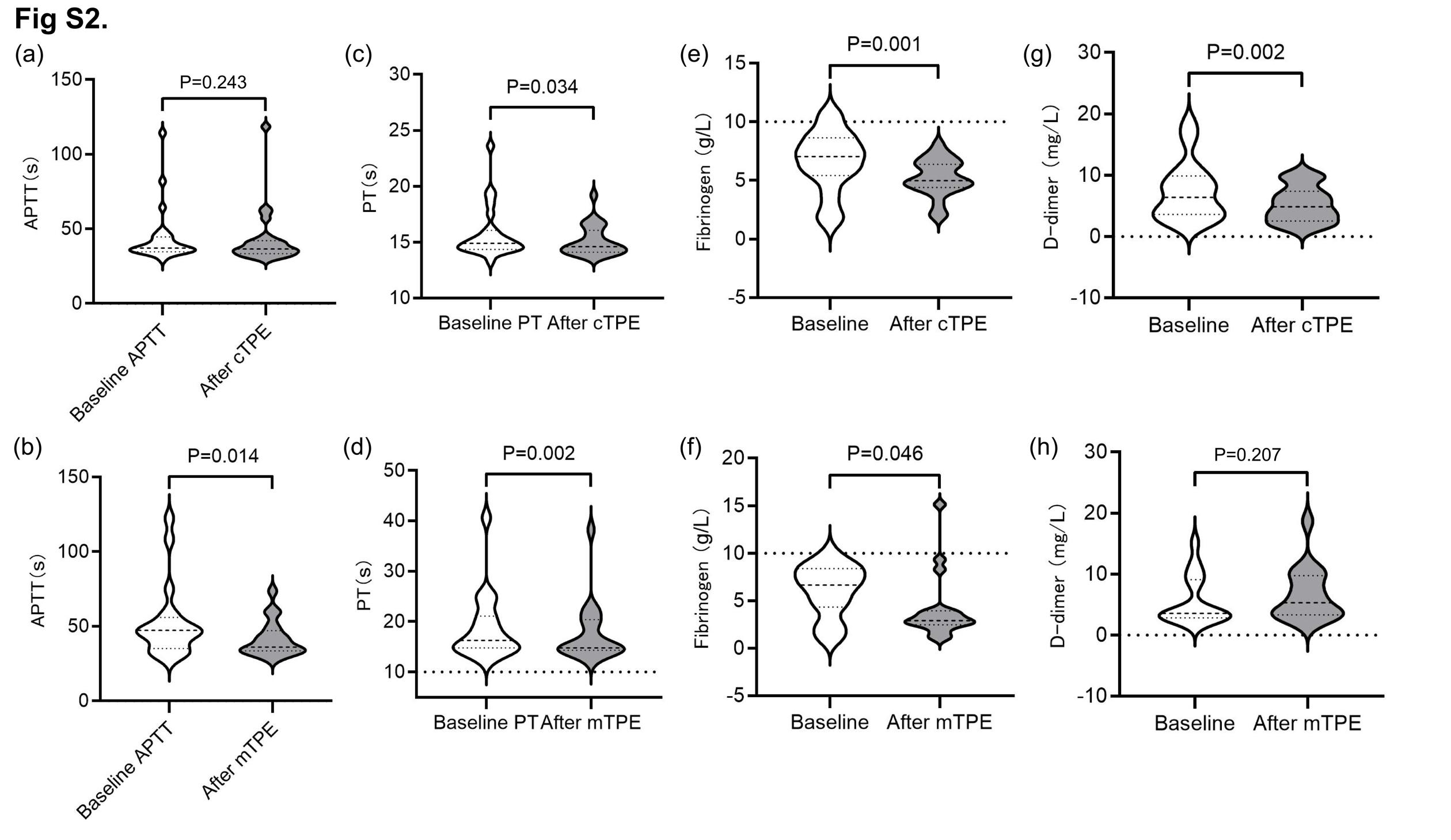

Supplement: SUPPLEMENTARY FIGURE S2 — Effects of different TPE modes on coagulation function indicators. (a,b) Change in APTT after two different modes of TPE; (c,d) change in PT after two different modes of TPE; (e,f) Changes in fibrinogen levels after two different modes of TPE; (g,h) changes in D-dimer levels after two different modes of TPE. ns, no significant difference. [file Image_2.jpeg]

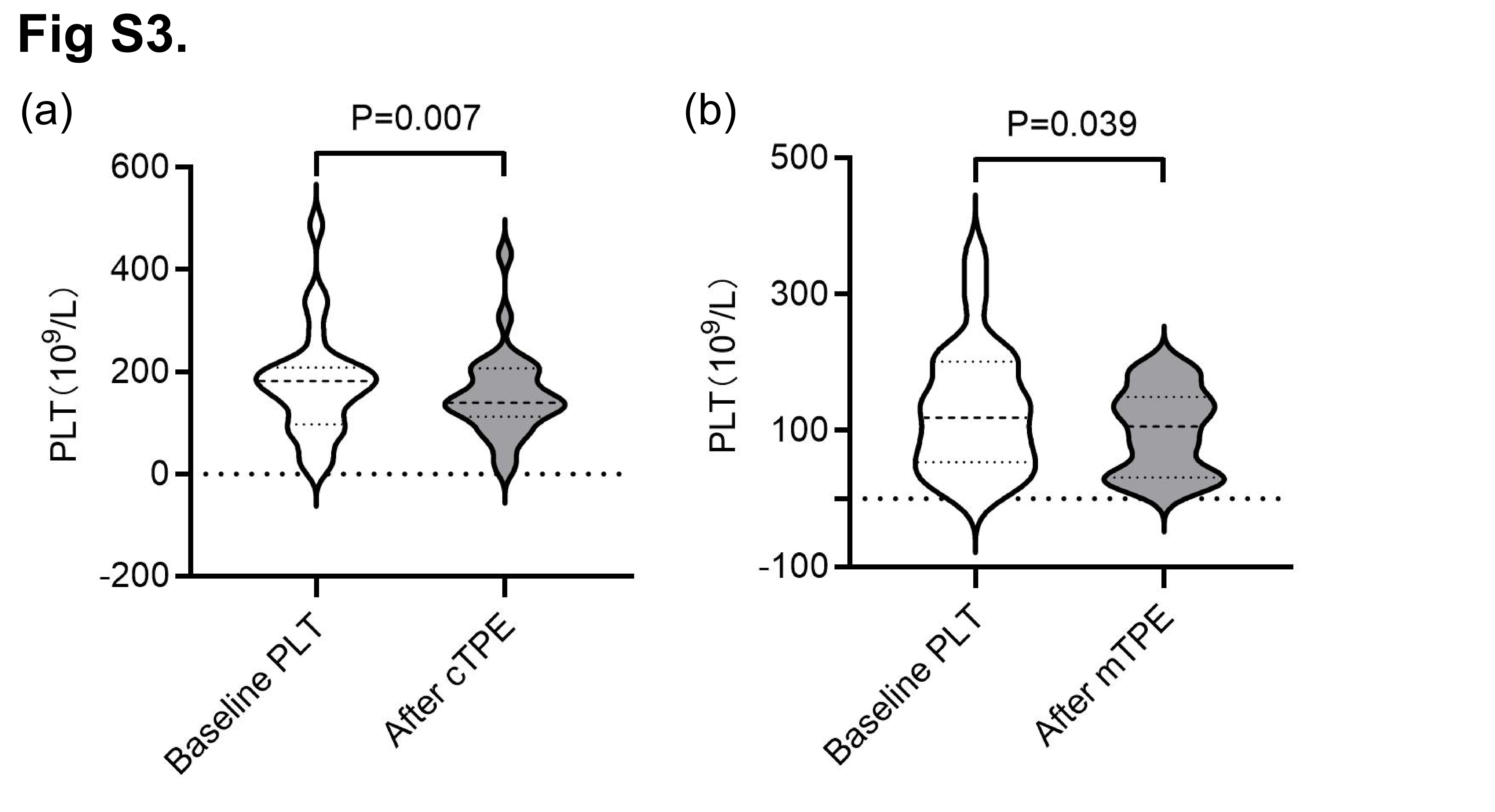

Supplement: SUPPLEMENTARY FIGURE S3 — Effects of different TPE modes on platelet levels. (a) Changes in platelet levels after cTPE treatment; (b) changes in platelet counts after mTPE treatment. [file Image_3.jpeg]

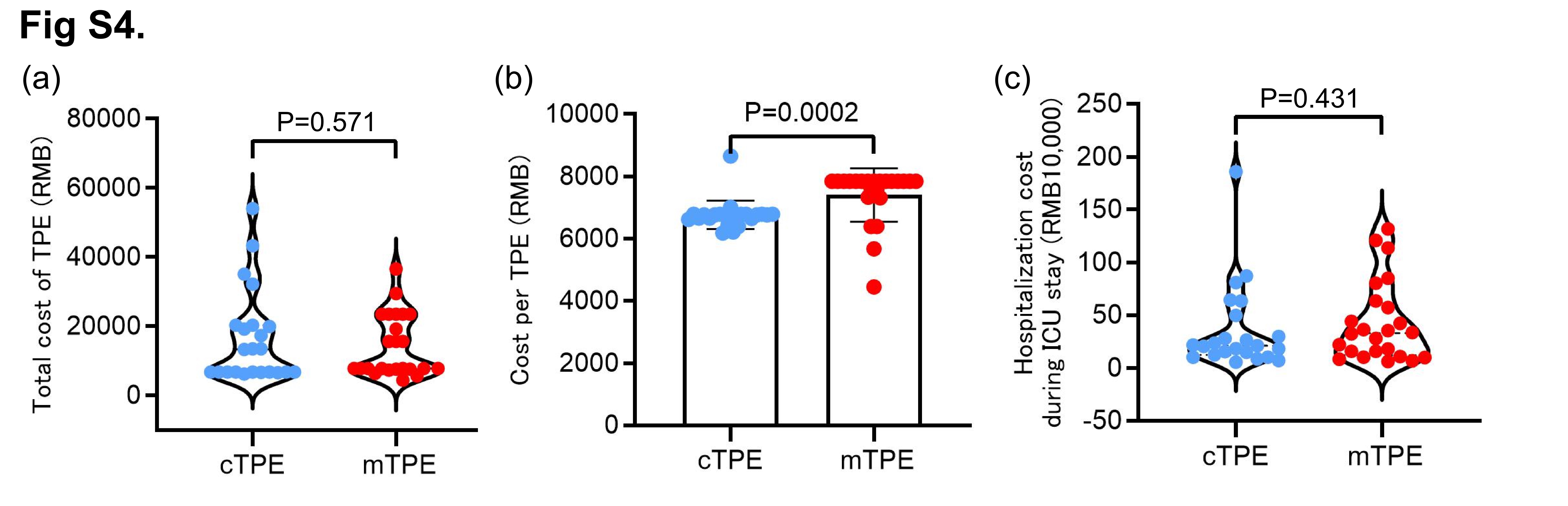

Supplement: SUPPLEMENTARY FIGURE S4 — The benefit comparison of different TPE modes. (a) Total cost comparison of different TPE modes; (b) single cost comparison of different TPE modes; (c) hospitalization cost comparison during ICU stay of different TPE modes. ns, no significant difference. [file Image_4.jpeg]

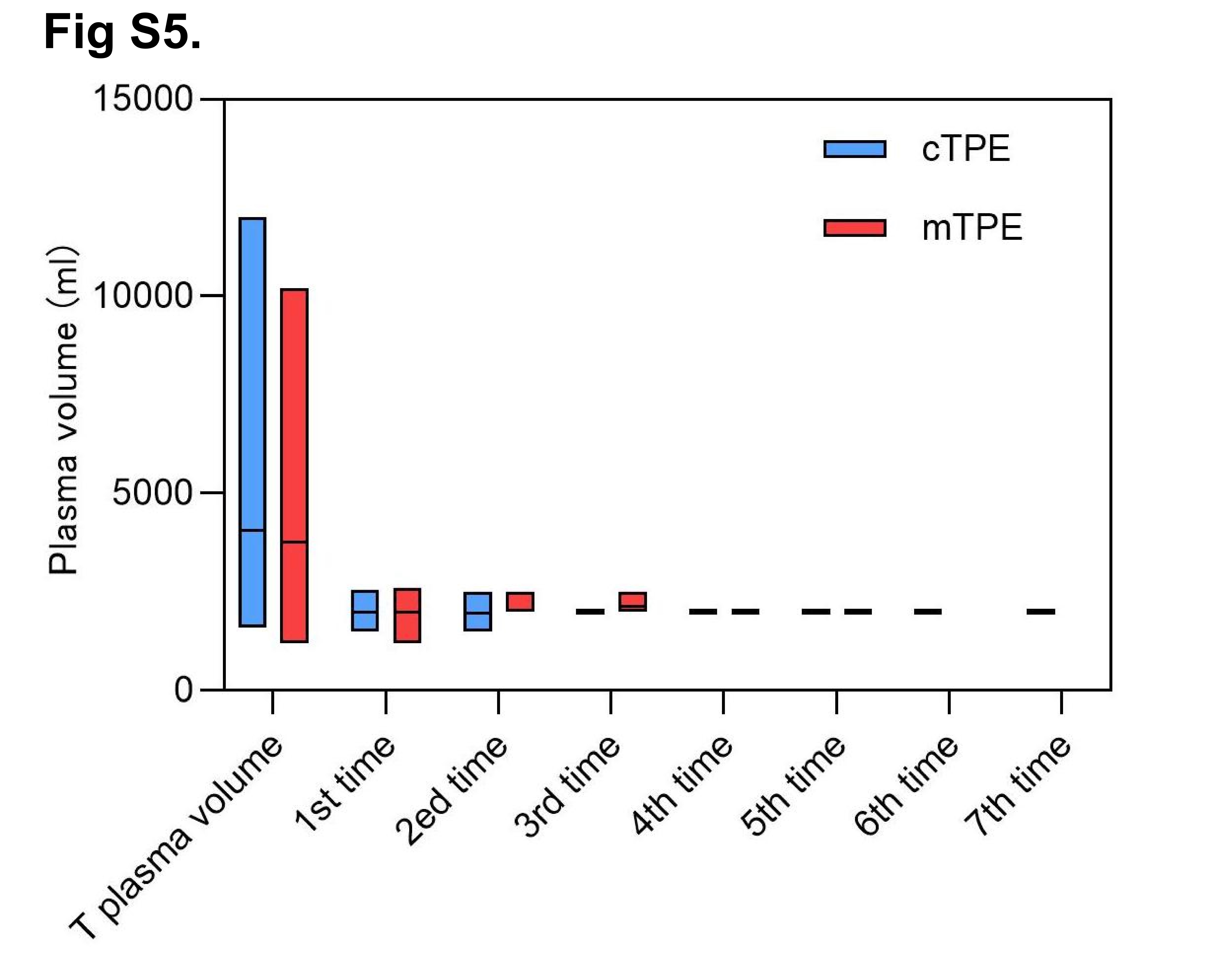

Supplement: SUPPLEMENTARY FIGURE S5 — Comparison of total plasma volume and single plasma volume in different TPE modes. [file Image_5.jpeg]
